# Addressing Intimate Partner Violence Through a Multifaceted Approach

**DOI:** 10.1002/hsr2.70873

**Published:** 2025-05-22

**Authors:** Dalmacito A. Cordero

**Affiliations:** ^1^ Department of Theology and Religious Education (DTRE) De La Salle University Manila Philippines

To the Editor,

I read with interest a recent article published in this journal regarding domestic violence during pregnancy. The authors aimed to compare the maternal–fetal attachment and fertility motivation in pregnant women with and without experience of violence. Their findings revealed that domestic violence during pregnancy affects the bond between mother and fetus, as well as fertility choices. Thus, they concluded that reducing domestic violence can enhance maternal–fetal attachment and improve fertility rates [1]. I firmly support this claim regarding the detrimental effects of violence not only to pregnant women, but to all women in general, especially the ones done at homes or the domestic ones, and the so‐called intimate partner violence (IPV). With this, I aim to shed light on the issue of IPV by presenting some global data and enumerating strategies to address the serious issue.

IPV refers to any behavior by an intimate partner or ex‐partner that causes physical, sexual, or psychological harm, including physical aggression, sexual coercion, psychological abuse, and controlling behaviors [2]. In Northeast India, different forms of IPV were experienced by 8766 married women which had adversely impacted various problematic reproductive health and pregnancy outcomes—6.9% had experienced non‐live births, 4.2% miscarriages, 2.4% abortions, 0.3% stillbirths, 16.9% terminated pregnancies, and 2.1% sexually transmitted infections (STI) [3]. The alarming issue is not isolated in India but is being experienced worldwide. There is an estimated 736 million women—almost one in three—have been subjected to physical and sexual intimate partner violence, non‐partner sexual violence, or both at least once in their lives (30% of women aged 15 and older) [4]. IPV victims can contribute to the onset of psychopathological conditions or can exacerbate mental health conditions, but on the other hand, existing mental health problems can increase vulnerability and predisposition to partner violence [5].

Several studies suggest a single or combined strategy to address IPV, but I suggest a multifaceted approach. Healthcare providers play a vital role through screening, offering ongoing support, and reviewing available prevention and referral options. Remote or in‐person screening of all patients at various times is needed because some women do not disclose abuse the first time they are asked. It should be done at periodic intervals, including during obstetric care. For ongoing support and referral options, clinicians must assist the patient in developing a safety plan for the patient, considering especially the risk factors (having experienced previous acts of violence, estrangement from a partner, threats to life, threats with a weapon, previous nonfatal strangulation, and partner access to a gun. Patients should be offered information that includes community resources (mental health services, crisis hotlines, rape relief centers, shelters, legal aid, and police contact information) and appropriate referrals [6].

The different institutions in the society can play a significant role in collaboration with the medical sector. Schools must strategically craft their respective values formation curriculum to strengthen their programs that will enhance the moral formation of students, especially focusing on antiviolence policies and responsibility. This is where the various student organizations will be most effective, with the support of the various offices. The government must untiringly promote public information campaigns to prevent violence, whether onsite or online. It can set up more helpdesk centers that are accessible to everyone, where victims can make reports when needed. Lastly, the Church should strengthen the administration of pre‐cana seminars and other preparatory activities before wedding celebrations for future couples. Constantly reminding couples of their vows of fidelity and rejecting any forms of abuse towards their partners is a big help for a violence‐free relationship.

## Author Contributions

**Dalmacito A. Cordero Jr.:** conceptualization, writing – original draft, methodology, writing – review and editing.

## Data Availability

The authors have nothing to report.
